# Effect of Postnatal Epigallocatechin-Gallate Treatment on Cardiac Function in Mice Prenatally Exposed to Alcohol

**DOI:** 10.3390/antiox12051067

**Published:** 2023-05-09

**Authors:** Vicente Andreu-Fernández, Mariona Serra-Delgado, Laura Almeida-Toledano, Àgueda García-Meseguer, Melina Vieiros, Anna Ramos-Triguero, Concha Muñoz-Lozano, Elisabet Navarro-Tapia, Leopoldo Martínez, Óscar García-Algar, María D. Gómez-Roig

**Affiliations:** 1 Grup de Recerca Infancia i Entorn (GRIE), Institut d’Investigacions Biomèdiques August Pi i Sunyer (IDIBAPS), 08036 Barcelona, Spain; 2 Biosanitary Research Institute, Valencian International University (VIU), 46002 Valencia, Spain; 3 Institut de Recerca Sant Joan de Déu, 08950 Esplugues de Llobregat, Spain; 4 BCNatal, Barcelona Center for Maternal-Fetal and Neonatal Medicine, Hospital Sant Joan de Déu and Hospital Clínic, Universitat de Barcelona, 08950 Barcelona, Spain; 5 Department of Neonatology, Hospital Clínic-Maternitat, ICGON, IDIBAPS, BCNatal, 08028 Barcelona, Spain; 6 Department of Pediatric Surgery, Hospital Universitario La Paz, 28046 Madrid, Spain

**Keywords:** FASD-like mouse model, prenatal alcohol exposure (PAE), binge alcohol drinking pattern, moderate alcohol drinking pattern, heart, echocardiography, oxidative stress, apoptosis, natural antioxidants, epigallocatechin-3-gallate (EGCG)

## Abstract

Prenatal alcohol exposure affects the cardiovascular health of the offspring. Epigallocatechin-3-gallate (EGCG) may be a protective agent against it, but no data are available regarding its impact on cardiac dysfunction. We investigated the presence of cardiac alterations in mice prenatally exposed to alcohol and the effect of postnatal EGCG treatment on cardiac function and related biochemical pathways. C57BL/6J pregnant mice received 1.5 g/kg/day (Mediterranean pattern), 4.5 g/kg/day (binge pattern) of ethanol, or maltodextrin until Day 19 of pregnancy. Post-delivery, treatment groups received EGCG-supplemented water. At post-natal Day 60, functional echocardiographies were performed. Heart biomarkers of apoptosis, oxidative stress, and cardiac damage were analyzed by Western blot. BNP and Hif1α increased and Nrf2 decreased in mice prenatally exposed to the Mediterranean alcohol pattern. Bcl-2 was downregulated in the binge PAE drinking pattern. Troponin I, glutathione peroxidase, and Bax increased in both ethanol exposure patterns. Prenatal alcohol exposure led to cardiac dysfunction in exposed mice, evidenced by a reduced ejection fraction, left ventricle posterior wall thickness at diastole, and Tei index. EGCG postnatal therapy restored the physiological levels of these biomarkers and improved cardiac dysfunction. These findings suggest that postnatal EGCG treatment attenuates the cardiac damage caused by prenatal alcohol exposure in the offspring.

## 1. Introduction

Alcohol consumption during pregnancy negatively affects the health of the developing fetus. Fetal alcohol spectrum disorders (FASD) are a group of conditions that may occur when an individual is prenatally exposed to alcohol. The most severe of these conditions is fetal alcohol syndrome (FAS), characterized by facial dysmorphology, growth restriction, central nervous system abnormalities during fetal development [1,2,3,4], and congenital malformations, including congenital heart defects [4,5,6,7].

The severity of fetal damage due to prenatal alcohol exposure (PAE) depends on the dose and timing of maternal alcohol consumption, the stage of pregnancy, the nutritional condition of the mother-to-be, and maternal and fetal genetic background. As for alcohol consumption, the binge or acute alcohol drinking pattern (Bin) is defined as a blood alcohol concentration (BAC) ≥ 0.08 g/dL, which typically corresponds to five drinks in men or four in women over a period of around two hours [8]. This consumption pattern is associated with a higher probability of having a child with a FAS phenotype [9,10]. The moderate drinking pattern, defined as two drinks in men and one in women, usually during meals [8], is more common in Mediterranean countries where the prevalence of FAS is lower. Still, these moderate levels of PAE increase the risk of FASD in the offspring.

The consumption of alcohol during pregnancy may have long-term consequences on the cardiovascular health of the offspring [11]. Echocardiography allows evaluating cardiac function in animal models, facilitating the study of cardiac disorders after exposure to different teratogens or potential prenatal treatments. However, echocardiographic outcomes differ depending on the stage of development of the subjects. Echocardiographic assessment in PAE animal models shows thinning of the ventricular walls and reduction in the ejection fraction (EF) in mice offspring [12] and thickening of the anterior and posterior wall of the left ventricle (LV) in adult rats [13].

Prenatal treatment based on antioxidants (e.g., folic acid, betaine, glutathione, or N-acetylcysteine) has been tested against the deleterious effects of alcohol on fetal heart, and promising beneficial effects on PAE mice have been observed [14,15,16]. Epigallocatequin-3-gallate (EGCG) has been shown to have a protective effect against cardiovascular diseases associated with its antioxidant, anti-inflammatory, anti-cardiac hypertrophy, and anti-myocardial infarction activity in vitro and in animal models [17,18,19,20]. It has been suggested that EGCG is a protective agent against FASD, ameliorating fetal growth restriction and preventing FASD-related cognitive impairment [21,22,23,24]. Despite its promising effects on prenatal growth restriction and neurodevelopment, the effect of EGCG on PAE-related heart damage remains unknown. In this study, we analyzed the presence of cardiac alterations (e.g., myocardial injury and cardiac dysfunction) in FASD young adult mice after acute (Bin pattern) or moderate (Mediterranean pattern-Med) prenatal alcohol exposure, as well as the effects of postnatal EGCG treatment on cardiac function and apoptosis, oxidative stress, and cardiac damage biomarkers altered by PAE. 

## 2. Materials and Methods

### 2.1. Animals, Housing, and Ethical Statement

We purchased eight weeks old C57BL/6J mice (30 male and 90 female) from Charles River (Barcelona, Spain) and housed them in the animal facilities of Sant Joan de Déu Hospital (Barcelona, Spain). Animals remained under controlled environmental conditions (20–24 °C, 55 ± 10% relative humidity, 12 h light/dark cycle); all mice had ad libitum standard food and water. Procedures on the animals followed the recommendations provided in the ARRIVE guidelines for the care and use of experimental animals and EU Directive 2010/63/EU for animal experiments, approved by the Animal Experimental Ethics Committee (CEEA) of the University of Barcelona, and registered on the Generalitat de Catalunya, Departament de Territori i Sostenibilitat (3FF6ZD9TL).

**Figure 1:**
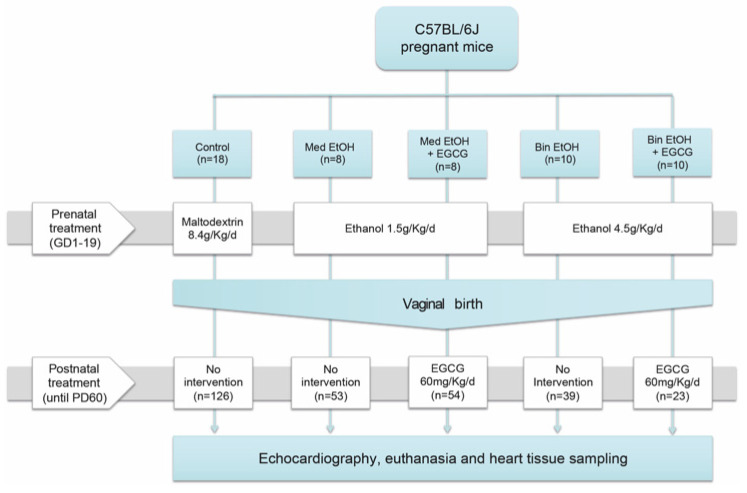
Experimental design. Alcohol and maltodextrin were given to study mice between Day 1 and Day 19 of pregnancy, which is equivalent to the first and second trimesters in human pregnancy. EGCG was administered after birth, from delivery (postnatal Day 1) until the end of the experiment (postnatal Day 60). Med: Mediterranean drinking pattern, EtOH: ethanol, Bin: binge drinking pattern, EGCG: epigallocatechin-3-gallate, *n*: number of individuals.

The five experimental groups were as follows (Figure 1):

(1) Control: mice were given maltodextrin solution (8.4 g/kg/day) between Day 1 and Day 19 of pregnancy by oral gavage and no postnatal treatment.
(2) Med alcohol (EtOH) pattern: mice were given 1.5 g/kg/day of ethanol between Day 1 and Day 19 of pregnancy by oral gavage and no postnatal treatment.
(3) Med EtOH pattern + EGCG: mice were given 1.5 g/kg/day of ethanol between Day 1 and Day 19 of pregnancy by oral gavage and postnatal EGCG at 60 mg/kg/day until pups were 60 days old.
(4) Bin EtOH pattern: mice were given 4.5 g/kg/day of ethanol between Day 1 and Day 19 of pregnancy by oral gavage and no postnatal treatment.
(5) Bin EtOH pattern + EGCG: mice were given 4.5 g/kg/day of ethanol between Day 1 and Day 19 of pregnancy by oral gavage and postnatal EGCG at 60 mg/kg/day until pups were 60 days old.

### 2.2. Alcohol Administration

Pure absolute ethanol was obtained from PanReac AppliChem ITW Reagents (Dublin, Ireland) and maltodextrin (Pure Series^®^) from Bulk Powders (Essex, UK). Ethanol and maltodextrin were administered by oral gavage [25] starting on Day 1 (presence of a sperm plug) until Day 19 of pregnancy; spontaneous delivery occurred between Day 19 and Day 21. The ethanol dose given to the Med EtOH and Med EtOH + EGCG groups was 1.5 g ethanol/kg/day in two administrations (eight-hour intervals); the Bin EtOH and Bin EtOH + EGCG groups were administered 4.5 g ethanol/kg/day once a day [21,26,27,28]. The control group received an isocaloric maltodextrin solution (8.4 g/kg/day), equivalent to the caloric intake of alcohol administered to the Bin group [21,29].

### 2.3. Postnatal Life and EGCG Administration

After birth, mice (mothers and pups) in the Bin EtOH + EGCG and Med EtOH + EGCG groups were given ad libitum EGCG-supplemented water [30,31] (Teavigo (94% EGCG) Green Tea, Healthy Origins, Pittsburgh, PA, USA) (twice a day to reduce EGCG oxidation). The average daily water intake [32] was 15 mL/100 g weight per day (estimated concentration of EGCG was 60 mg/kg/day) (Figure 1). 

EGCG was given to the pups from postnatal Day 1 until Day 60 (end of the experimental period); an echocardiography was then performed, and pups were sacrificed for cardiac tissue collection. 

Pups were kept with their mothers until postnatal Day 22, after which they were weaned and gender segregated. During the 22 days pups remained with their mothers, the newly-born mice not only received EGCG through the supplemented water, but also through breast milk, being safe if doses were not greater than 200 mg/kg/day as reported elsewhere [33,34].

### 2.4. Functional Echocardiography Performance

A VEVO 3100 (FUJIFILM Visual Sonics, Toronto, Canada) imaging system equipped with a transducer (22–55 MHz) (MX5550D, FUJIFILM Visual Sonics) was used to perform functional echocardiographies in young adult female offspring on Day 60 post-delivery. 

Mice were anesthetized with 2% isoflurane, delivered in 0.5 L/min 100% oxygen in the induction chamber, and maintained with 1.5% isoflurane, delivered with 0.5 L/min 100% oxygen via a nose cone. Systolic function was assessed from a parasternal short-axis view on M-mode to evaluate left ventricle internal dimensions at diastole (LVIDd) and systole (LVIDs) and calculate fractional shortening (FS) and EF [35]. Heart rate (HR) and LV posterior wall thickness at systole and diastole were also assessed at M-mode to next calculate LV posterior wall (PW) thickening (PWT). 

To evaluate the diastolic function, we used the trans-mitral inflow pulse-wave Doppler obtained in an apical 4-chamber and LV long-axis view measuring E and A waves, which allowed us to calculate the ratio of peak velocity of early to late filling of mitral flow (E/A). We also assessed the isovolumic contraction time, isovolumic relaxation time, and left ventricular ejection time, to calculate the Tei index [35,36,37].

### 2.5. Blood Alcohol Concentration

One milliliter of maternal blood was collected in heparin BD Vacutainer^®^ by cardiac puncture 45 min after the administration of alcohol (either at Med or Bin doses) or maltodextrin. Samples were maintained for five minutes at room temperature and centrifuged at 1750× *g* for 20 min at 4 °C. Blood alcohol concentrations (BAC) were determined by measuring absorbance at 570 nm in serum samples using the Ethanol Assay Kit (MAK076, Sigma-Aldrich, Sant Louis, MO, USA) following the indications of the manufacturer.

### 2.6. Western Blot Analysis

Whole protein extracts were obtained with a Polytron processor (Omni Tissue Homogenizer, Omni International, Kennesaw, GA, USA) by mechanically disrupting tissue samples in RIPA buffer (Life Technologies S.A, 89900, Carlsbad, CA, USA) for 30 s. Proteins were quantified with the DC Protein Assay kit (Bio-rad Laboratories S.A., Madrid, Spain), and absorbance was measured at 780 nm (Lowry test). Next, 40 µg of total protein were mixed with 6 µL of 5× loading buffer (3.125 mL 1M Tris-HCl (pH = 6.8), 5.75 mL glycerol 87%, 1 g SDS, 1 mL β-mercaptoethanol, and 1 mL 5% bromophenol blue). This mixture was heated at 95 °C on a thermoblock (Thermo Scientific, Waltham, MA, USA) to denaturalize the protein and then loaded (30 µL per well) in RIPA buffer. Electrophoresis was performed in running buffer (3.03 g/L of Tris Base, 1.44 g/L of glycine, and 1 g/L of SDS) using the molecular weight marker (precision plus protein dual color standard from BioRad, 1610374) on 7.5%, 10%, 12%, and 14% acrylamide gels. Polyvinylidene fluoride membranes (Bio-Rad Laboratories SA, 162-0177), first activated in methanol for five minutes, were used for protein transfer. The latter was conducted in transfer buffer (3.03 g/L of Tris-Base, 14.4 g/L of glycine, and 200 mL/L of methanol) at 4 °C for two hours at 400 mA or overnight at 240 mA, depending on the molecular weight of the protein. Next, three five-minute washes were performed with tris-buffered saline (TBS-T) (2.4 g/L Tris-HCl (pH = 7.6), 8.8 g/L NaCl, and 1 mL Tween 20). Membranes were covered for 30 min with 5% BSA diluted in TBS-T. Finally, the membranes were incubated overnight with the primary antibody (1:1000 dilutions in BSA 5%) at 4 °C with shaking. The following day, the primary antibody was removed by washing three times for five minutes with TBS-T. Next, membranes were embedded with the secondary anti-rabbit or anti-mouse antibody for two hours with shaking at room temperature. The Pierce ECLWB Substrate (Thermo Fisher Technologies (Waltham, MA, USA) was used to develop the membranes in an iBright CL1000 device (Thermo Fisher Scientific, Barcelona, Spain) in a dark room. The intensity of the bands was determined by densitometric analysis using the Image J program. Values quantified by densitometry were normalized using control proteins.

### 2.7. Antibodies

Glycogen synthase kinase-3 beta (Gsk3β) (ref. ab227208, 46 kDa) and B-type natriuretic peptide (BNP) (ref. ab236101, 15 kDa) were obtained from Abcam (Madrid, Spain); Nrf2 (ref. af3925, 90 kDa) from R&D systems (Minneapolis, MN, USA); the hypoxia inducible factor 1-Alpha (Hif1-α) (ref. sc-13515, 130 kDa), β-cell lymphoma 2 (Bcl-2) (ref. sc-7382), glutathione peroxidase (GPx) (ref. sc-133160), catalase (ref. sc-271803), and troponin I (ref. sc-133117, 29 kDa) from Santa Cruz Biotechnology, Inc. (Dallas, TX, USA); superoxide dismutase 2 (SOD-2) (ref.13141) and Bcl-2-like protein 4 (Bax) (ref. 2771) were from Cell Signaling (Danvers, MA, USA); alpha-tubulin (ref. T8203, dilution 1:2000, 50 kDa) and anti-rabbit IgG secondary antibody (ref. A0545, dilution 1:2000) from Sigma-Aldrich (Sant Louis, MO, USA); goat anti-mouse IgG (ref. G21040, dilution 1:10,000) from Thermo Fisher Technologies (Waltham, MA, USA).

### 2.8. Statistical Analyses

For statistical analyses, the SPSS v.22 (IBM, Chicago, IL, USA) and GraphPad 6.0 (Prism, San Diego, CA, USA) software were used. Descriptive statistics are presented as mean and standard deviation (SD). Inter-group comparisons were performed with the non-parametric Kruskal-Wallis test (Dunn’s correction for multiple comparisons) to assess the differences in protein expression in heart tissue. Statistical significance was set at *p* < 0.05 * for all analyses (*p* < 0.01 **; *p* < 0.001 ***; *p* < 0.0001 ****). The experiments were repeated at least three times to obtain the mean for each sample and at least eight different samples from different litters were used for the statistical analyses.

## 3. Results

Bin or Med alcohol doses (4.5 g/kg/day or 1.5 g/kg/day, respectively) or maltodextrin (8.4 g/kg/day) were administered to study mice during pregnancy. After delivery, EGCG-supplemented (60 mg/kg/day) water or EGCG-free water was given to the mice from Day 1 to Day 60 post-delivery.

Total number of young adult mice (offspring) was 295 (Figure 1). Fourteen pregnant mice from the Bin group had no viable offspring or their pups died over the first days of life (until Day 22); similar results have been reported elsewhere [38,39,40].

**Figure 2:**
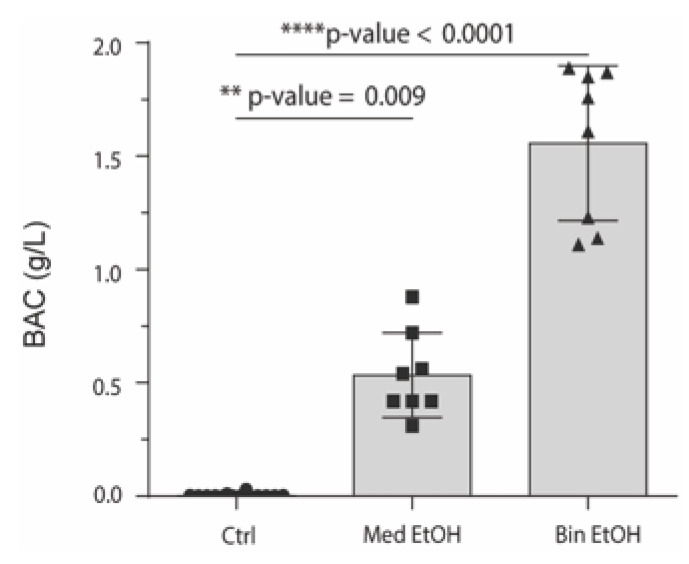
Blood alcohol concentrations in female mice post-administration of alcohol under different experimental conditions. Med EtOH: 30% of ethanol solution (1.5 g/kg/day in two administrations); Bin EtOH: 40% of ethanol solution (4.5 g/kg/day); Ctrl: control; Med: Mediterranean drinking pattern, Bin: binge drinking pattern; EtOH: ethanol; BAC: blood alcohol concentration. ▲: binge drinking pattern, ■: Mediterranean drinking pattern, ●: control.

Maternal blood was collected to determine BACs after the mice were given ethanol, which showed the differences between the control group (*n* = 14) and the Med EtOH (*n* = 8) and Bin EtOH (*n* = 8) groups (Figure 2). Mean BAC was 0.53 g/L, 95% CI [0.3776; 0.6899] and 1.56 g/L, 95% CI [1.271; 1.844] for the Med and Bin groups, respectively. For both groups, mean BACs were in agreement with the definitions by the Centers for Disease Control and Prevention and the National Institute on Alcohol Abuse and Alcoholism (NIAAA) for Bin and Med human-like drinking patterns [8]. 

### 3.1. Analysis of Cardiac Biomarkers

**Figure 3:**
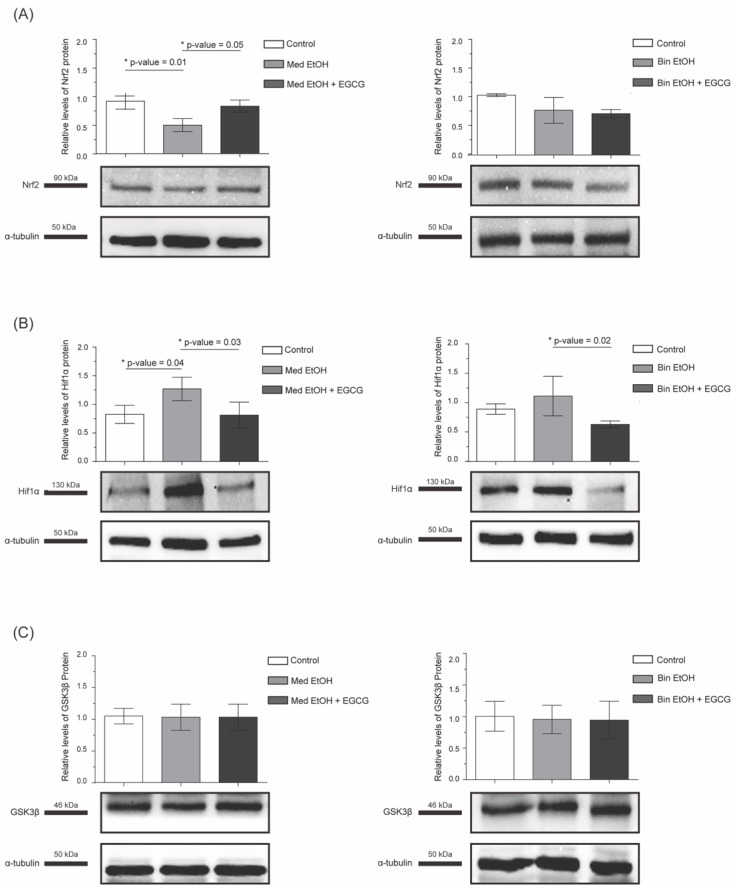
Nrf2 (**A**), Hif1-α (**B**), and GSK3β (**C**) levels in heart tissue mice to analyze oxidative stress and apoptosis after parental alcohol exposure (two different patterns of alcohol exposure, Med and Bin). Effect of postnatal treatment with EGCG on oxidative stress and apoptosis biomarkers. Nrf2: nuclear factor erythroid-2-related factor 2; Gsk3β: glycogen synthase kinase-3 beta; Hif1-α: hypoxia inducible factor 1-alpha; Med: Mediterranean group; EtOH: ethanol, Bin: binge group, EGCG: epigallocatechin-3-gallate.

**Figure 4:**
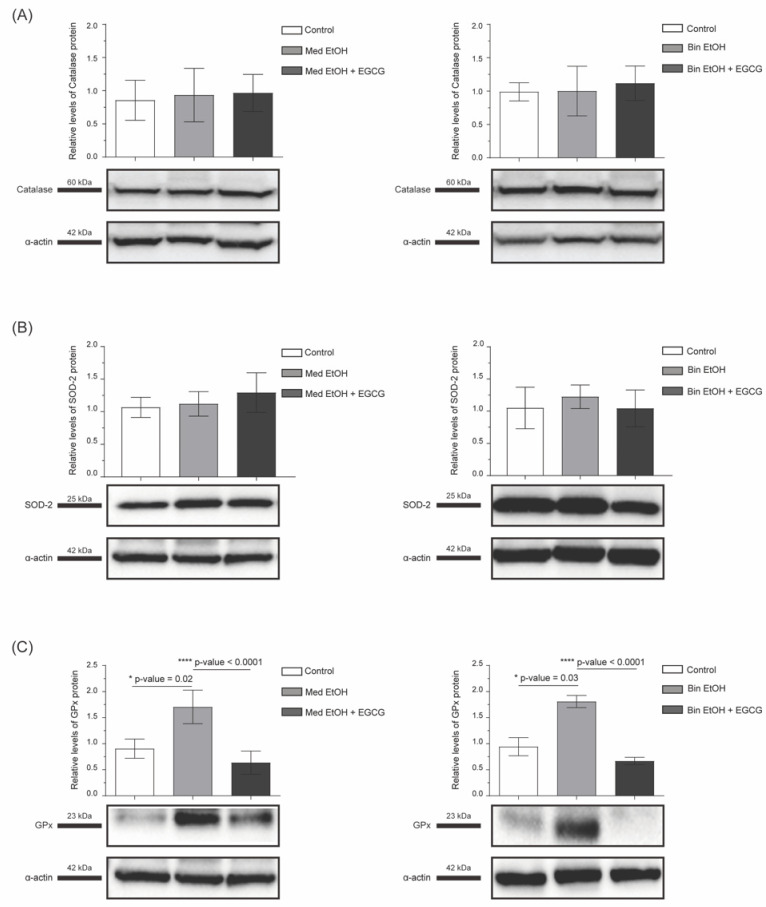
Catalase (**A**), SOD-2 (**B**), and GPx (**C**) levels in heart tissue of mice to analyze oxidative stress after parental alcohol exposure (two different patterns of alcohol exposure, Med and Bin). Effect of postnatal treatment with EGCG on oxidative stress biomarkers. SOD-2: superoxide dismutase 2; GPx: Glutathione peroxidase; Med: Mediterranean group; EtOH: ethanol, Bin: binge group, EGCG: epigallocatechin-3-gallate.

To analyze oxidative stress we assessed Nrf2, Hif1-α, catalase, superoxide dismutase 2 (SOD-2), and glutathione peroxidase (GPx). There was a significant decline (*p* = 0.01) of the Nrf2 transcription factor in mice prenatally exposed to the Med pattern and a partial recovery to control levels in the Med EtOH + EGCG group. No statistically significant differences were determined for Nrf2 between the control, Bin EtOH, and Bin EtOH + EGCG groups (Figure 3A). There was an increase of Hif1-α in PAE heart tissue compared to control tissue, being statistically significant (*p* = 0.04) for the Med group. Recovery of Hif1-α physiological level was seen for the Med EtOH + EGCG and Bin EtOH + EGCG groups (Figure 3B). GPx expression in heart tissue significantly increased in PAE groups (*p* = 0.02 in the Med drinking group, *p* = 0.03 in the Bin drinking group), and postnatal EGCG therapy led to the recovery of the normal values in both groups (*p* < 0.0001) (Figure 4C). No intergroup differences were found for SOD-2 and catalase (Figure 4A,B). 

**Figure 5:**
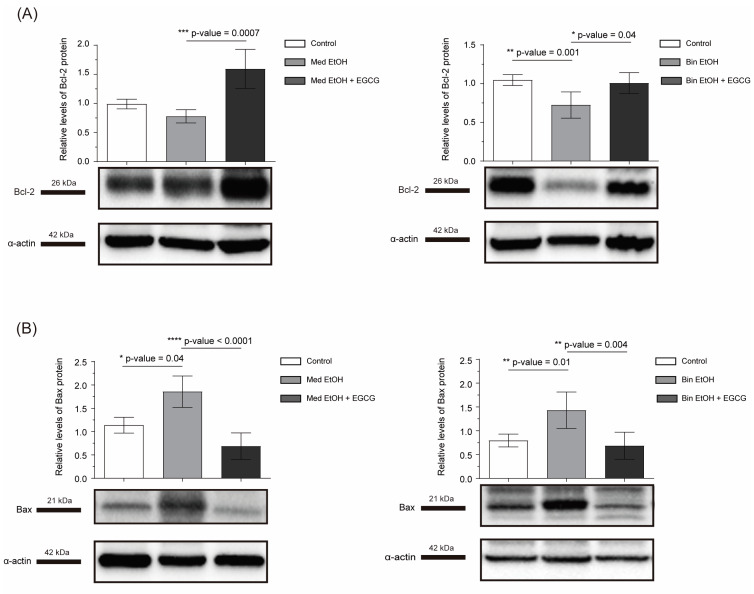
Bcl-2 (**A**) and Bax (**B**) levels in heart tissue of mice to analyze apoptosis after parental alcohol exposure (two different patterns of alcohol exposure, Med and Bin). Effect of postnatal treatment with EGCG on apoptosis biomarkers. Bcl-2: B-cell lymphoma 2, Bax: Bcl-2-like protein 4, Med: Mediterranean group; EtOH: ethanol, Bin: binge group, EGCG: epigallocatechin-3-gallate.

To analyze apoptosis we assessed Gsk3β, Bax, and Bcl-2. No intergroup differences were found for Gsk3β (Figure 3C). Bcl-2 expression is downregulated in the Bin PAE drinking pattern (*p* = 0.001); postnatal EGCG treatment upregulated it in animals from both prenatal alcohol exposure patterns (Figure 5A). Bax increased in heart tissue of PAE mice in Med (*p* = 0.04) and Bin (*p* = 0.01) drinking patterns (Figure 5B), and postnatal EGCG treatment downregulated it to control levels.

**Figure 6:**
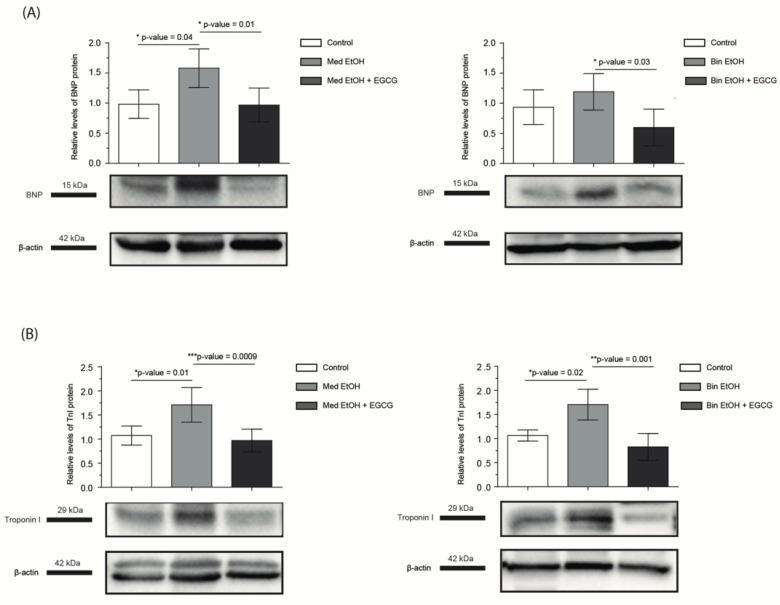
B-type natriuretic peptide (**A**) and troponin I (**B**) levels in infant mice to assess heart biomarkers after parental alcohol exposure (two different patterns of alcohol exposure, Med and Bin). Effect of postnatal treatment with EGCG on cardiac biomarkers. Trop I: troponin I; BNP: B-type natriuretic peptide; Med: Mediterranean drinking pattern; EtOH: ethanol; Bin: binge drinking pattern; EGCG: epigallocatechin-3-gallate.

An increase of the specific cardiac markers BNP (Figure 6A) and troponin I (Figure 6B) was seen in the PAE groups; the increase was statistically significant for BNP in the Med EtOH group. EGCG administration decreased BNP to a control level in PAE mice (Figure 6A). Increased levels of troponin I were found for the Med EtOH and Bin EtOH groups compared to controls, which returned to control levels in groups treated with EGCG (Figure 6B).

### 3.2. Echocardiographic Analysis

**Figure 7:**
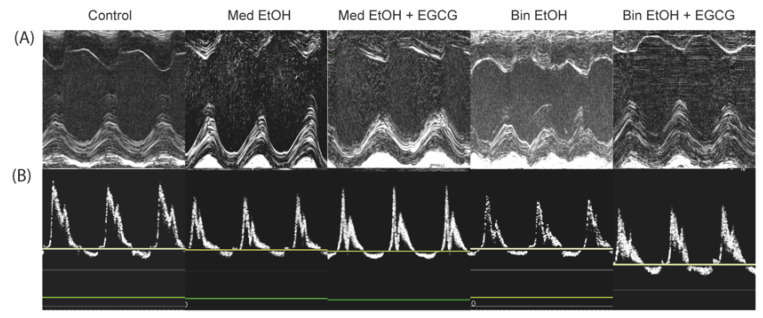
Echocardiographic images from mice with different patterns of prenatal alcohol exposure -Mediterranean (Med EtOH) or binge (Bin EtOH) -, compared with controls and prenatal alcohol exposure plus postnatal epigallocatechin-3-gallate treatment (Med EtOH + EGCG) and (Bin EtOH + EGCG). (**A**): Representative M-mode images on parasternal short-axis view to assess systolic function of the heart of experimental mice; (**B**): Representative images of pulse-wave Doppler of mitral inflow to assess diastolic cardiac function in experimental mice. Med: Mediterranean drinking pattern; EtOH: ethanol, Bin: binge drinking pattern; EGCG: epigallocatechin-3-gallate.

Representative results of systolic (Figure 7A) and diastolic (Figure 7B) heart function evaluated by echocardiography for each offspring experimental group. The results in Table 1 include 89 echocardiographies: 33 from the control group, 16 from the Med EtOH group, 16 Med from the EtOH + EGCG group, 17 from the Bin EtOH group, and seven from the Bin EtOH + EGCG group.

**Figure 8:**
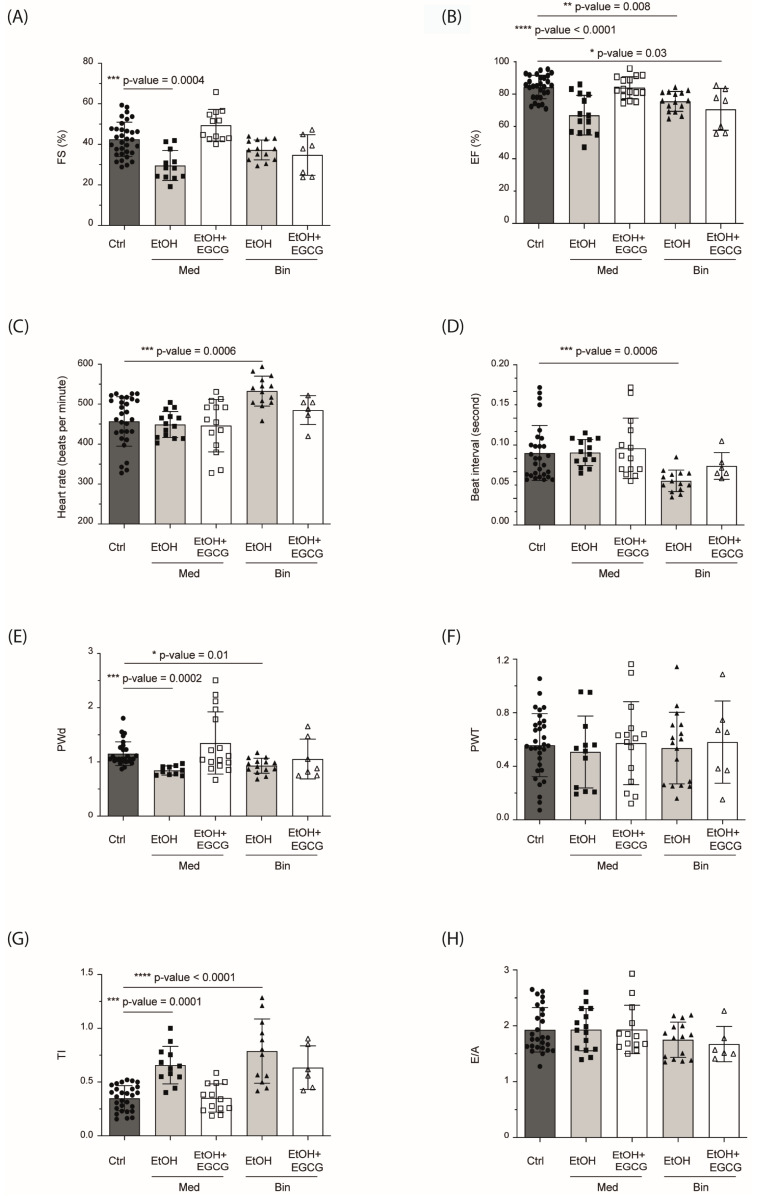
Cardiac function variables measured by echocardiography in mice exposed to two patterns of alcohol consumption and treated with EGCG. Each value is represented by a specific symbol. Statistical analyses were performed using the nonparametric Kruskal Wallis with Dunn’s correction. (**A**) Fractional shortening (%), (**B**) Ejection fraction, (**C**) Cardiac frequency, (**D**) t1 beat, (**E**) Left ventricle posterior wall thickness at diastole, (**F**) Left ventricular wall thickening, (**G**) Tei index, (**H**) Ratio of peak velocity of early to late filling of mitral flow (E/A). Symbols: ● Control; Mediterranean (■) or binge (▲) alcohol consumption pattern; EGCG treatment of the Mediterranean (□) or binge (△) alcohol consumption pattern mice. LV: left ventricle; Ctrl: control; Med: Mediterranean drinking pattern, EtOH: ethanol; Bin: binge drinking pattern; EGCG: epigallocatechin-3-gallate.

FS was lower in the Med EtOH group compared to the control group (Figure 8A), (Table 1). The EF decreased in PAE mice (Figure 8B). Treatment with EGCG reestablished the control phenotype in the Med group (Table 1). Heart rate significantly increased (*p* = 0.0006) in mice in the Bin EtOH group (Figure 8C,D). As for the thickness of the posterior wall of the LV at diastole, a significant reduction (*p* = 0.0002 in the Med group; *p* = 0.01 in the Bin group) was found in PAE groups compared to the controls (Table 1) and recovery in the Med EtOH + EGCG group (Figure 8E). 

No significant differences in left ventricular posterior wall thickening were found between PAE groups and controls (Figure 8F). PAE groups were associated with an increase in the pulsed Doppler Tei index compared to the control groups. The Tei index of PAE + EGCG specimens showed no statistical differences when compared to the control group, indicating a clear recovery of altered values by PAE (Figure 8H). The Tei index increased in PAE animals at the expense of the isovolumic contraction time (IVCT) and isovolumic relaxation time (IVRT). Animals treated with EGCG had similar IVCT values to that of controls. Similarly, the IVRT of mice in the Bin group treated with EGCG after birth was similar to that of the control group.

## 4. Discussion

Med and Bin PAE during fetal development induces oxidative stress and causes damage to the heart that persists into early adulthood. Cardiac dysfunction in prenatally exposed young adult mice is restored with the administration of EGCG after birth, particularly in mice exposed to the Med pattern.

Alcohol is primarily broken down in the liver by the enzymes alcohol dehydrogenase and aldehyde dehydrogenases. High BAC due to Bin drinking saturates this metabolic pathway, in which case cytochrome P450 also comes into play; this alternative pathway induces the production of reactive oxygen species (ROS), promoting cardiac damage and apoptosis [41] and consequently many of the effects seen in FASD syndrome [25]. As to maternal Med drinking pattern, little is known about the associated molecular mechanism; however, even low or moderate levels of PAE increase the risk of FASD in the offspring. The antioxidant defense system (endogenous and exogenous antioxidants) prevents ROS-associated damage. SOD, GPx, and catalase are endogenous antioxidants that reduce the production of ROS by enzymatic strategies [42]. Their expression in FAS individuals depends on the tissue and stage of development when the assessment is made. In the brain of fetal mice, the expression of these antioxidant enzymes is decreased [43]. Contrarily, no differences are found in the fetal liver and the placenta in comparison to the control group [43]. GPx activity is also reduced in the hippocampus of rats at postnatal Day 28, but SOD activity is increased and no differences are seen for catalase [44]. The analysis of these enzymatic antioxidants in the cardiac tissue of mice embryos (gestational Day 8.5) exposed to alcohol, shows a reduction of SOD and GPx in the alcohol group [45]. However, in heart tissue from mice at postnatal Day 60, SOD, GPx, and catalase expression are upregulated [46]. Accordingly, our results reveal an increased GPx expression (Figure 4C), but no differences in SOD-2 or catalase (Figure 4A,B). A potential transcriptional activation of antioxidant enzymes [46] in an attempt to reduce alcohol-induced cardiac injury occurs during adulthood, while fetal mechanisms to respond to cardiac damage may be insufficient. The antioxidant effect of EGCG alone can compensate for oxidative stress produced by PAE, so the activation of the endogenous antioxidant system is unrequired.

Med and Bin alcohol drinking patterns reduce Nrf2 expression in cardiac tissue (Figure 3A) as found in placentas from a PAE rat model [47]. Postnatal EGCG therapy in Med individuals, upregulates Nrf2 expression (Figure 3A), modulating the induction of antioxidant response against oxidative damage probably by inactivating KEAP 1 [48,49]. Contrarily, there are no changes in Bin exposed individuals with postnatal EGCG treatment, probably because its antioxidant effect cannot compensate for the massive damage caused by ROS. Previous studies have shown different effects of alcohol on Hif1-α depending on the drinking pattern and examined tissue [50]. Our data demonstrate how PAE induces Hif1-α expression in the heart (Figure 3B) in response to hypoxia, which may alter cardiomyocytes [51]. EGCG treatment restores the physiological levels of Hif1-α (Figure 3B) as has been described elsewhere in tissues such as skin and nasal polyp fibroblasts [52,53]. 

Regarding apoptosis, alcohol exposure induces downregulation of the anti-apoptotic Bcl-2 protein (Figure 5A) and increases the activity of the pro-apoptotic Bax in individuals exposed to the Bin drinking pattern (Figure 5B). This has been shown in the embryonic heart, where Bcl-2 is downregulated, while the effector caspase 3 (degrades intracellular proteins by proteolysis, mediating cellular death) is upregulated [54]. After EGCG treatment, Bcl-2 expression increases (Figure 5A) in both prenatal ethanol-exposed groups, reducing Bax expression and blocking apoptosis (Figure 5B). This pathway has been already explored by evaluating the use of EGCG after myocardial ischemia, and the findings were similar [55].

As a response to myocardial damage, BNP [46] and troponin I are increased in cardiomyocytes of young adult mice prenatally exposed to alcohol in the two human-like drinking patterns (Figure 6). EGCG downregulates BNP [20] and troponin I cardiac expression (Figure 6). Cardiomyocytes and cardiac fibroblasts synthetize pro-BNP, which is split into BNP and NT-pro-BNP. BNP is a biologically active molecule [56]. Its production and secretion are stimulated by cardiac wall stress and silent myocardial damage [57]. In the normal heart, the atria are the main producer of BNP, however, when there is chronic cardiac mechanical stress, heart ventricles increase BNP production [58]. The increase in ventricular BNP correlates with its increase in plasma [59], used as a biomarker of heart failure in clinical practice. The endocrine function of BNP, characterized by diuretic, natriuretic, and vasorelaxant properties, is to alleviate cardiac injury [60]. BNP also has a paracrine effect on the heart, aimed at reducing fibrosis and hypertrophy [61,62].

Troponin I regulates the state of thin and thick filaments in the sarcomere [63], inhibiting muscle contraction in the absence of calcium. The slow skeletal muscle cardiac troponin I isoform is predominant in the developing fetal heart, replaced by the mature sarcomere cardiac isoform at around birth [64]. In congenital heart defects, there is a delay in the expression of the latter isoform [65]. Alcohol-associated cardiac tissue damage results in the increase of troponin I (Figure 6B) as seen in young adult diabetic rats [66]. Some authors report that cardiac expression of troponin I decreases in older mice compared to younger individuals [67]. These findings suggest that troponin I cardiac expression may change throughout life. In clinical practice, troponin I serum levels are assessed to determine cardiac damage. When myocyte damage occurs, there is release of troponin I into the systemic circulation, although to date there is no data on the relationship with its expression in the heart. BNP and troponin I plasma levels increase in adults with chronic alcoholism, which suggests alcoholic cardiomyopathy. Future studies should compare the levels of troponin I, BNP, and NT-pro-BNP in serum and cardiac tissue and evaluate their expression throughout life. 

Echocardiographic images have shown that alcohol intake during pregnancy is associated with cardiovascular disorders [7,68]. PAE impairs systolic function (reduces FS and EF). Similar results have been reported in a study with a prenatal mice model in which the study subjects received 2.9 g/kg intraperitoneal ethanol injections at gestational days 6.75 and 7.25 [12,69]. Our results show that PAE-related systolic dysfunction persists into adult life with Med and Bin human-like drinking patterns. Figure 8A,B shows significant decreases of FS (*p* = 0.0004) and EF (*p* < 0.0001) in the Med group and a decrease of EF (*p* = 0.008) in the Bin group with a trend towards a decrease of FS. In our study, administration of postnatal EGCG prevents systolic dysfunction in cases of low alcohol exposure (Figure 8A,B), which is similar to the results obtained in a study that used EGCG therapy in a mouse heart failure model produced by the aortic arch ligation [20]. Acute alcohol consumption affects the autonomic control of the heart due to the increase in basal HR of the most affected mice, as has been shown for human infants, for whose baseline heart rate was 4.6 bpm higher in infants prenatally exposed to alcohol in comparison to the control group [70,71]. This increase may be caused by changes in the autonomic nervous system with reduced parasympathetic activity [72]. Physiological values are achieved with postnatal EGCG therapy (Figure 8C). However, in line with our findings, chronic intake of low alcohol doses during pregnancy in a rat model (ad libitum liquid diet 6% vol/vol) showed no HR changes in female mice [73].

Cardiac remodeling due to human-like Med and Bin alcohol consumption patterns characterized by myocardial thinning is evidenced by a decrease in LV PW diastole (Figure 8E). This finding is supported by studies in which hematoxylin-eosin histological analysis in chick embryos [37] and echocardiographic evaluation of neonatal mice hearts [12] show a reduction in ventricular wall thickness in PAE animals. This alteration has been also seen in chronic adult ethanol drinkers with alcoholic cardiomyopathy, characterized by an increased left ventricular mass, wall thinning, left ventricular dilatation, and ventricular dysfunction [74]. We performed no pathomorphological studies in our study. However, in a study carried out with alcoholic rats, correlations were found between the reduction in the thickness of the left ventricular wall, the decrease in EF and FS, and the dilatation of the cardiac cavities (assessed by echocardiography) with morphologic findings in the anatomy of the heart (bifocal dilatation of the cardiac ventricles and fatty infiltration of the myocardium) [75]. Furthermore, another study carried out with PAE mice, which evaluated cardiac left ventricular indexes at postnatal Day 60, observed mild cardiac hypertrophy in PAE animals [46]. Nevertheless, echocardiographic assessment in an adult PAE rat model showed a thickening of cardiac ventricular walls [13]. Cardiomyocyte damage, evidenced by the increase of cardiac BNP and troponin I (Figure 6), results in the thinning of the ventricular wall in newly born and young mice, while in adult rats an increased deposition of interstitial collagen induces fibrosis of the damaged heart tissue leading to LV thickness in adulthood [13]. Lowering of cardiac damage biomarkers post-EGCG treatment also promotes cardiac remodeling. Considering our findings, future research should address the anatomy and pathology of the heart, evaluate cardiac tissue changes under these morphologic alterations, and compare the results with those of animals postnatally treated with EGCG.

We used the myocardial performance index to assess early-life cardiac dysfunction produced by alcohol intake during fetal development. The Tei index is higher in both PAE groups compared to controls. The beneficial effects of postnatal EGCG therapy on global cardiac dysfunction in PAE mice are observed not only in systolic function, but also in the improvement of diastolic function as has been previously shown in a mouse model of restrictive cardiomyopathy where EGCG produced an acceleration in sarcomere relaxation and calcium breakdown in myocardial cells [76].

Fetal echocardiography is a validated diagnostic tool for alcohol-related congenital heart defects. The detection of early changes at cellular and molecular levels, which may provide information on future cardiac dysfunction, is a challenge for clinicians. Oxidative stress (SOD-2, GPx, catalase, Nrf2, and Hif1-α), apoptosis (Bcl-2 and Bax), and cardiac damage (BNP and troponin I) biomarkers may be useful tools for the early detection of patients with a suspected diagnosis of FASD at risk of cardiac dysfunction. Future studies are necessary to evaluate the sensitivity and specificity of these biomarkers in humans. In addition, functional echocardiographic studies will allow premature detection of cardiac remodeling and functional disorders during childhood or early adulthood in individuals at risk, allowing physicians to implement prevention strategies and novel therapies.

Different study groups have tested prenatal treatments for alcohol-related cardiac damage in animal models. Folic acid, betaine, and glutathione were tested on animals exposed to alcohol during gastrulation, resulting in the recovery of normal gene expression and improved embryonic heart function [14,15,16]. Prenatal administration of N-acetylcysteine has positive effects on the hearts of PAE mice; it corrects alcohol-induced changes in heart collagen and improves cardiac function [69]. In an animal model, prenatal administration of EGCG attenuated ethanol-induced oxidative stress and apoptosis, ameliorating fetal growth restriction and preventing FASD-related cognitive impairment [21,22,23,24]. EGCG administration in children with Down’s Syndrome, has shown promising results in its safety and efficacy [77,78]. The pharmacokinetics and oral bioavailability of EGCG have been explored, showing an oral bioavailability of around 26.5% [79] in mice and between 1.6–4.95% in rats [80]. Furthermore, repeated doses of catechin intake modulates its bioavailability, upregulating the intestinal EGCG transporter [81] and improving its safety profile [82,83,84]. Teavigo^®^ has been demonstrated to have a better bioavailability profile than other EGCG preparations [85]. Similar or higher EGCG doses have been previously used in humans [86,87] and animal models [88,89,90,91] confirming its safety. According to the European Food Safety Agency, the toxicity level in humans is 800 mg/day or above [92].

The promising results on postnatal EGCG antioxidant therapy we show in this study should encourage future research focusing on clinical trials aimed at evaluating cardiac function in children with FASD and the beneficial effects of EGCG. 

Our study uses an FASD-like mice model to reduce the influence of confounding and environmental factors present in human studies. One of the main limitations of this study is the alcohol exposure of mice in the first and second-trimester human-equivalent, but not in the third. HR is a modifying factor on Doppler-derived diastolic indexes [93]; despite of this, the Tei index does not seem to be affected by it [94]. Finally, the use of a specific dose of isoflurane, which may lead to variations of mean FS values, hamper the comparison with other studies with different protocols to anesthetize the mice [35]. However, it does not alter the statistical differences between our experimental groups.

## 5. Conclusions

Our study shows the detrimental effects of PAE on the offspring´s heart regardless of the drinking pattern, acute (Bin) or moderate (Med). To the best of our knowledge, this study demonstrates for the first time that postnatal administration of EGCG to the newly born during infancy and adolescence restores cardiac expression biomarkers and echocardiographic parameters in animals exposed to either human-like drinking patterns. EGCG firmly positions itself as a potential therapeutic agent to improve FASD cardiac-related effects.

## Figures and Tables

**Table 1:** Descriptive values of cardiac function variables measured by echocardiography in mice exposed to two alcohol consumption patterns (Mediterranean and binge) and treated with epigallocatechin-3-gallate.

Echocardiographic Variables	Experimental Groups	*n*	Mean	Standard Deviation	Standard Error	95% Confidence Interval		Minimum	Maximum
						Lower Limit	Upper Limit		
Left ventricular internal dimension at diastole (LVIDd)	Control	33	4.546	0.663	0.115	4.311	4.781	3.157	5.678
	Med EtOH	12	4.556	0.895	0.258	3.987	5.125	2.204	5.441
	Med EtOH + EGCG	12	4.389	0.693	0.200	3.949	4.829	3.312	5.785
	Bin EtOH	14	4.660	0.670	0.179	4.273	5.047	3.795	6.410
	Bin EtOH + EGCG	7	4.705	0.501	0.189	4.242	5.169	4.225	5.745
Left ventricular internal dimension at systole (LVIDs)	Control	33	2.624	0.597	0.104	2.413	2.836	1.455	3.629
	Med EtOH	12	3.212	0.735	0.212	2.745	3.679	1.578	4.044
	Med EtOH + EGCG	12	2.243	0.610	0.176	1.855	2.631	1.458	3.323
	Bin EtOH	14	2.939	0.584	0.156	2.601	3.276	2.232	4.332
	Bin EtOH + EGCG	7	3.060	0.493	0.186	2.604	3.516	2.229	3.479
Fractional shortening (FS) (%)	Control	33	42.513	8.486	1.477	39.504	45.522	28.825	59.340
	Med EtOH	12	29.575	7.380	2.131	24.885	34.264	19.160	41.932
	Med EtOH + EGCG	12	49.433	7.813	2.255	44.469	54.397	40.098	65.754
	Bin EtOH	14	37.266	4.922	1.315	34.424	40.107	29.424	44.032
	Bin EtOH + EGCG	7	34.797	10.001	3.780	25.548	44.046	23.772	47.243
Ejection fraction (EF) (%)	Control	29	84.366	0.072	0.013	81.609	87.124	70.948	95.488
	Med EtOH	14	66.977	0.122	0.032	59.902	74.052	47.171	86.084
	Med EtOH + EGCG	15	84.025	0.067	0.017	80.273	87.776	74.451	95.984
	Bin EtOH	15	75.515	0.062	0.016	72.094	78.936	64.846	84.318
	Bin EtOH + EGCG	7	70.601	0.129	0.049	58.625	82.576	55.705	85.316
LV posterior wall thicknesses at systole (PWs) (mm)	Control	32	1.777	0.469	0.083	1.608	1.946	0.956	3.028
	Med EtOH	12	1.476	0.600	0.173	1.095	1.857	0.944	3.046
	Med EtOH + EGCG	15	1.969	0.665	0.172	1.601	2.337	1.094	3.865
	Bin EtOH	17	1.548	0.278	0.068	1.405	1.691	1.238	2.208
	Bin EtOH + EGCG	7	1.596	0.339	0.128	1.282	1.910	1.131	2.019
LV posterior wall thicknesses at diastole (PWd) (mm)	Control	28	1.054	0.219	0.041	1.067	1.237	0.868	1.807
	Med EtOH	11	0.833	0.082	0.261	0.783	0.901	0.743	0.972
	Med EtOH + EGCG	16	1.076	0.573	0.143	1.044	1.655	0.669	2.506
	Bin EtOH	14	0.940	0.138	0.037	0.850	1.010	0.683	1.171
	Bin EtOH + EGCG	7	0.882	0.367	0.139	0.715	1.395	0.748	1.656
LV posterior wall thickening (PWT) (%)	Control	32	55.719	23.492	4.153	47.250	64.189	7.280	105.470
	Med EtOH	12	50.715	26.815	7.741	33.677	67.752	19.206	95.570
	Med EtOH + EGCG	15	57.288	30.999	8.004	40.122	74.455	12.189	116.284
	Bin EtOH	17	53.593	26.763	6.491	39.832	67.353	16.067	114.348
	Bin EtOH + EGCG	7	58.035	30.691	11.600	29.650	86.419	15.157	108.690
Beat interval (seg)	Control	29	0.134	0.021	0.004	0.126	0.142	0.114	0.183
	Med EtOH	14	0.134	0.010	0.003	0.129	0.140	0.119	0.149
	Med EtOH + EGCG	14	0.137	0.023	0.006	0.124	0.151	0.113	0.183
	Bin EtOH	14	0.113	0.008	0.002	0.108	0.118	0.101	0.131
	Bin EtOH + EGCG	6	0.124	0.010	0.004	0.114	0.135	0.115	0.143
Heart rate (HR) (bpm)	Control	29	456.910	61.584	11.436	433.484	480.335	327.869	526.316
	Med EtOH	14	448.759	32.689	8.736	429.885	467.633	402.685	504.202
	Med EtOH + EGCG	14	446.234	65.407	17.481	408.469	483.999	327.869	530.973
	Bin EtOH	14	532.799	37.528	10.030	511.131	554.467	458.015	594.059
	Bin EtOH + EGCG	6	484.995	36.146	14.756	447.062	522.927	419.580	521.739
E wave velocity (cm/s)	Control	27	0.131	0.197	0.038	0.053	0.209	0.028	1.040
	Med EtOH	15	0.069	0.040	0.010	0.047	0.091	0.033	0.171
	Med EtOH + EGCG	13	0.105	0.072	0.020	0.062	0.149	0.045	0.266
	Bin EtOH	15	0.092	0.063	0.016	0.057	0.126	0.035	0.242
	Bin EtOH + EGCG	6	0.078	0.075	0.031	-0.002	0.157	0.041	0.231
A wave velocity (cm/s)	Control	27	0.068	0.096	0.018	0.030	0.106	0.019	0.500
	Med EtOH	15	0.036	0.020	0.005	0.025	0.047	0.018	0.079
	Med EtOH + EGCG	13	0.058	0.047	0.013	0.030	0.086	0.024	0.162
	Bin EtOH	15	0.053	0.036	0.009	0.034	0.073	0.019	0.134
	Bin EtOH + EGCG	6	0.048	0.049	0.020	-0.003	0.099	0.026	0.147
Ratio of peak velocity of early to late filling of mitral inflow (E/A)	Control	27	1.928	0.398	0.077	1.771	2.085	1.273	2.651
	Med EtOH	15	1.932	0.378	0.098	1.722	2.141	1.397	2.600
	Med EtOH + EGCG	13	1.934	0.433	0.120	1.673	2.196	1.500	2.933
	Bin EtOH	15	1.752	0.315	0.081	1.577	1.926	1.353	2.194
	Bin EtOH + EGCG	6	1.675	0.316	0.129	1.344	2.006	1.414	2.269
Isovolumic contraction time (IVCT) (% Cardiac cycle (CC))	Control	24	6.260	1.394	0.284	5.671	6.848	3.529	8.333
	Med EtOH	13	8.043	1.028	0.285	7.422	8.664	6.250	10.16
	Med EtOH + EGCG	8	6.977	1.747	0.617	5.516	8.437	5.072	9.836
	Bin EtOH	14	8.790	1.862	0.498	7.715	9.864	5.723	13.36
	Bin EtOH + EGCG	5	6.150	1.422	0.636	4.385	7.916	3.922	7.692
Isovolumic relaxation time (IVRT) (% CC)	Control	20	7.662	1.458	0.3260	6.980	8.345	5.303	10.86
	Med EtOH	14	12.96	3.488	0.932	10.94	14.97	8.271	18.45
	Med EtOH + EGCG	11	10.59	2.361	0.712	9.002	12.17	6.780	13.86
	Bin EtOH	12	11.27	2.370	0.684	9.761	12.77	8.621	15.49
	Bin EtOH + EGCG	5	8.464	2.502	1.119	5.358	11.57	4.724	10.78
Left ventricular ejection time (LVET) (ms)	Control	28	0.057	0.017	0.003	0.050	0.063	0.038	0.107
	Med EtOH	12	0.051	0.011	0.003	0.044	0.058	0.028	0.067
	Med EtOH + EGCG	13	0.062	0.014	0.004	0.054	0.071	0.040	0.091
	Bin EtOH	12	0.044	0.011	0.003	0.037	0.052	0.031	0.068
	Bin EtOH + EGCG	6	0.040	0.007	0.003	0.032	0.048	0.034	0.051
Tei index (TI)	Control	2	0.350	0.117	0.022	0.305	0.395	0.155	0.521
	Med EtOH	13	0.658	0.174	0.050	0.547	0.768	0.403	1.000
	Med EtOH + EGCG	8	0.352	0.131	0.036	0.273	0.432	0.187	0.586
	Bin EtOH	12	0.788	0.299	0.086	0.599	0.978	0.419	1.286
	Bin EtOH + EGCG	5	0.635	0.203	0.083	0.422	0.847	0.422	0.905

Med: Mediterranean drinking pattern, EtOH: ethanol, Bin: binge drinking pattern, EGCG: epigallocatechin-3-gallate.

## Data Availability

All data are included in the article.
